# Coupling Modern Portfolio Theory and Marxan enhances the efficiency of Lesser White-fronted Goose’s (*Anser erythropus*) habitat conservation

**DOI:** 10.1038/s41598-017-18594-2

**Published:** 2018-01-09

**Authors:** Jie Liang, Xiang Gao, Guangming Zeng, Shanshan Hua, Minzhou Zhong, Xiaodong Li, Xin Li

**Affiliations:** 1 College of Environmental Science and Engineering, Hunan University, Changsha, 410082 PR China; 2 Key Laboratory of Environmental Biology and Pollution Control (Hunan University), Ministry of Education, Changsha, 410082 PR China

## Abstract

Climate change and human activities cause uncertain changes to species biodiversity by altering their habitat. The uncertainty of climate change requires planners to balance the benefit and cost of making conservation plan. Here optimal protection approach for Lesser White-fronted Goose (LWfG) by coupling Modern Portfolio Theory (MPT) and Marxan selection were proposed. MPT was used to provide suggested weights of investment for protected area (PA) and reduce the influence of climatic uncertainty, while Marxan was utilized to choose a series of specific locations for PA. We argued that through combining these two commonly used techniques with the conservation plan, including assets allocation and PA chosing, the efficiency of rare bird’s protection would be enhanced. In MPT analyses, the uncertainty of conservation-outcome can be reduced while conservation effort was allocated in Hunan, Jiangxi and Yangtze River delta. In Marxan model, the optimal location for habitat restorations based on existing nature reserve was identified. Clear priorities for the location and allocation of assets could be provided based on this research, and it could help decision makers to build conservation strategy for LWfG.

## Introduction

Climate change affects the distribution and abundance of endangered species and may result in habitat loss, habitat fragmentation and other disturbances^1^. In order to conserve habitat quality for endangered species, actions, including developing nature reserves, should be urgently undertaken^2^. However, funding limitations and risk of investment are two issues of significant importance in conserving or building nature reserves^3^ for Lesser White-fronted Goose (LWfG), a species sensitive to habitat environment change, especially. The conservation plan should be focused on making better use of the investment^4^. This issue forces decision-makers to allocate the investment in a highly efficient way. Therefore, identifying essential regions and selecting the optimal portfolio for LWfG’s conservation investment is necessary^5,6^.

To solve the problem of LWfG’s population decline, biological scientists have proposed several measurements. One of the approaches is analyzing spatial distribution on the local level using species distribution model (SDM or habitat suitability model (HSM))^7^. However, it is unsatisfactory to use such results to allocate investments for LWfG^8^. One reason is that the the uncertainty existing in estimating climate parameters, which makes it difficult to implement existing conservation plan^9^. It is too complex for scientists to make definitive climate change predictions. For example, the Coupled Model Intercomparison Project Phase 5 (CMIP5) presents 4 Representative Concentration Pathways (RCP) scenarios of 21st century greenhouse-gas emissions^10^. These scenarios are based on 19 different models and a range of assumptions. Underlying this fact is the risk of climate uncertainty attaches to the future outcomes of current conservation investments. The other reason is that the results of HSM can not reflect suitability of building nature reserve^11^. Selecting the locations of nature reserve is also relative to the cost and return. For instance, many protected areas are located in sites of relatively low economic and biodiversity values^12^. Therefore, these areas do not sustain an adequate representation of habitat suitability index (HSI), and the question that whether decision makers invest more funds here needs to be questioned.

Modern portfolio theory (MPT) is a regional investment tool by utilizing information about covariance to explicit targeting of management investments^13^. It applies joint probability distribution of outcomes for all possible assets in a portfolio to select efficiently investing methods that reduce the influence of risk^13,14^. By spreading around exposure risk to decrease the impact of a bad performance asset, the MPT reflects an idea of risk diversification. Researches on cost-effective wetland habitat conservation usually use the climate variables and bioclimatic variables as influencing factors of conservation^15^. However, climate change uncertainty makes it difficult to estimate the species’ probability distribution in future scenarios^16,17^. When future climate change is different from what we expected, the opportunity cost to decision makers in conservation plan will change as well. Assigning an appropriate portfolio to the asset using MPT can cope with this problem. Through choosing portfolio weights correctly, efficient portfolios can be found in this scenario for a given level of return^18^.

Since we have found the portfolio weights to meet our requirements, the challenge of minimizing planning conservation area with high protection value can be set. Marxan is a systematic conservation planning approach to identify the optimal locations by simulated annealing algorithm^19^, which means the cost of reserve construction is minimized on the basis of achieving representativeness and persistence^20,21^. It tries to find a near-optimal solution that achieves the predetermined conservation goals while keeping the cost of planning units as low as possible. It has been applied extensively over the world^22^. Different from MPT analyses, Marxan chooses the prior area for conservation but haven’t assigned the resource weights to invest^23^.

**Figure 1:**
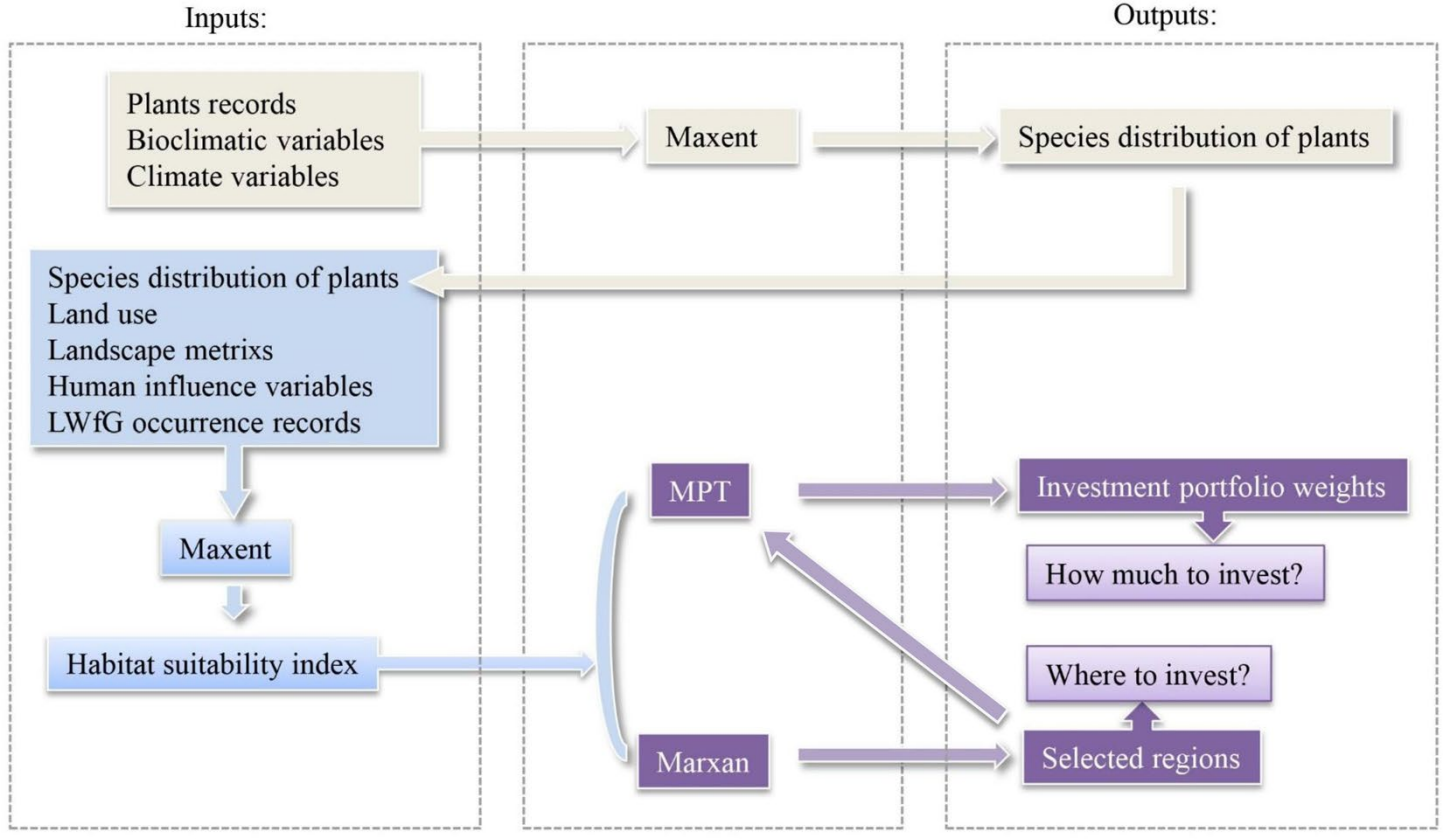
Flowchart of the approach to couple MPT and Marxan.

In this paper, we used Marxan and MPT to characterize optimal investing method and spatial target of LWfG’s habitat protection planns (Fig. 1). Firstly, we built habitat suitability models for LWfG using maximum entropy approach (Maxent), and the model result was HSI. Subsequently, we used HSI and existing nature reserves data to run Marxan models, and identified the minimum locations to develop nature reserve for LWfG’s protection. Then, HSI was employed as a benefit of the expected return to find optimal investment portfolios using MPT. The results could be used to guide LWfG’s habitat investment in low risk. Finally, a targeted result that met the conservation objective could be drawn by using the results of MPT analyses and Marxan.

## Results

### Habitat suitability models

The high AUC values (>0.8) (Supplementary Table S1) for all models indicated that our models could reasonably capture the relationship between LWfG and plant, and thus these models could be could be used to project the habitat suitability of LWfG species. As determined by jackknife analysis of Maxent (Supplementary Table S2 and Fig. S1), HSI of *Carex heterolepis, Alopecurus aequalis* and *Eleocharis migoana* were the most important variables. This finding was consistent with previous research^24,25^ that quality and abundance of foraging plants profoundly affected LWfG distributions. The response curve of 10 variables was illustrated in Supplementary Fig. S2. Most curves symbolized a rising trend.

**Figure 2:**
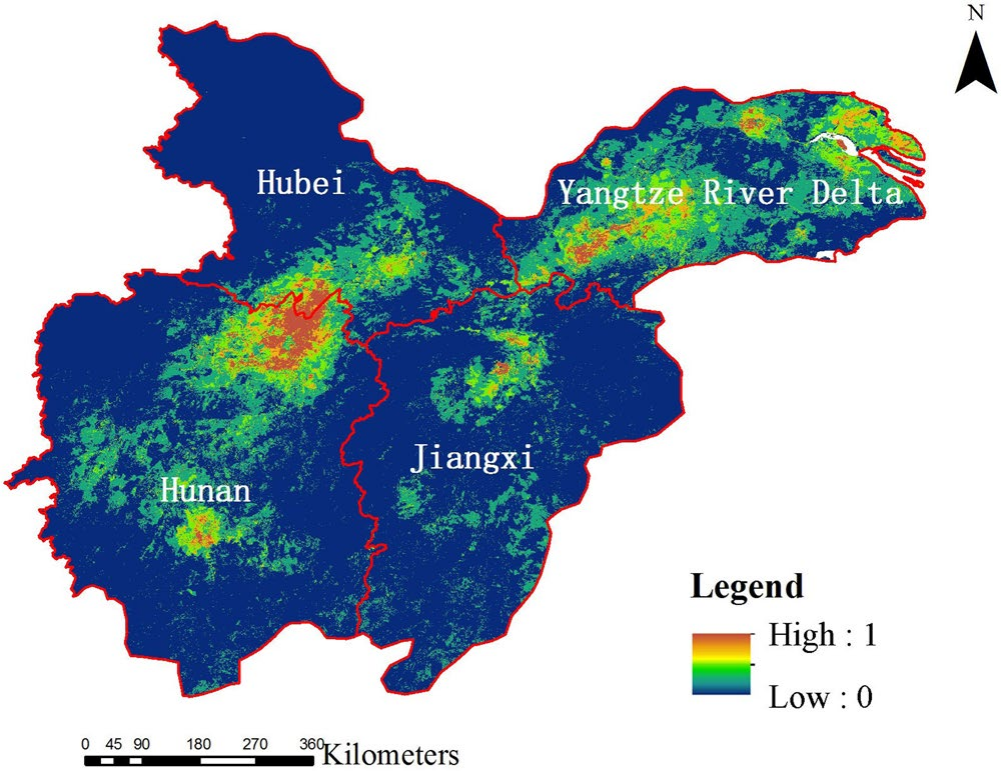
Distribution of habitat suitability of LWfG in the current scenario in accordance to Maxent model. The map was plotted using ArcGIS 10.2 (ESRI, Redlands, CA, USA, http://www.esri.com/).

Table 1 listed the average HSI for four climate scenarios (Fig. 2). Obviously, climate change would dramatically reduced the habitat quality of LWfG. Minimum value for current scenarios (0.451) was even larger than maximum value for future scenarios (0.444). Using a threshold of minimum training presence, area with HSI higher than the threshold was appointed as the suitability habitat for LWfG. Comparing to current scenarios, the proportion of suitable habitats in the future scenarios will also decrease (Supplementary Fig. S3).

**Table 1:** Average value of HSI value in the current and future scenarios for LWfG.

Region	Current	RCP 2.6	RCP 4.5	RCP8.5
Hunan	0.582	0.404	0.444	0.367
Hubei	0.534	0.276	0.233	0.189
Yangtze River delta	0.477	0.355	0.395	0.421
Jiangxi,	0.451	0.370	0.394	0.349

### PA optimization

**Figure 3:**
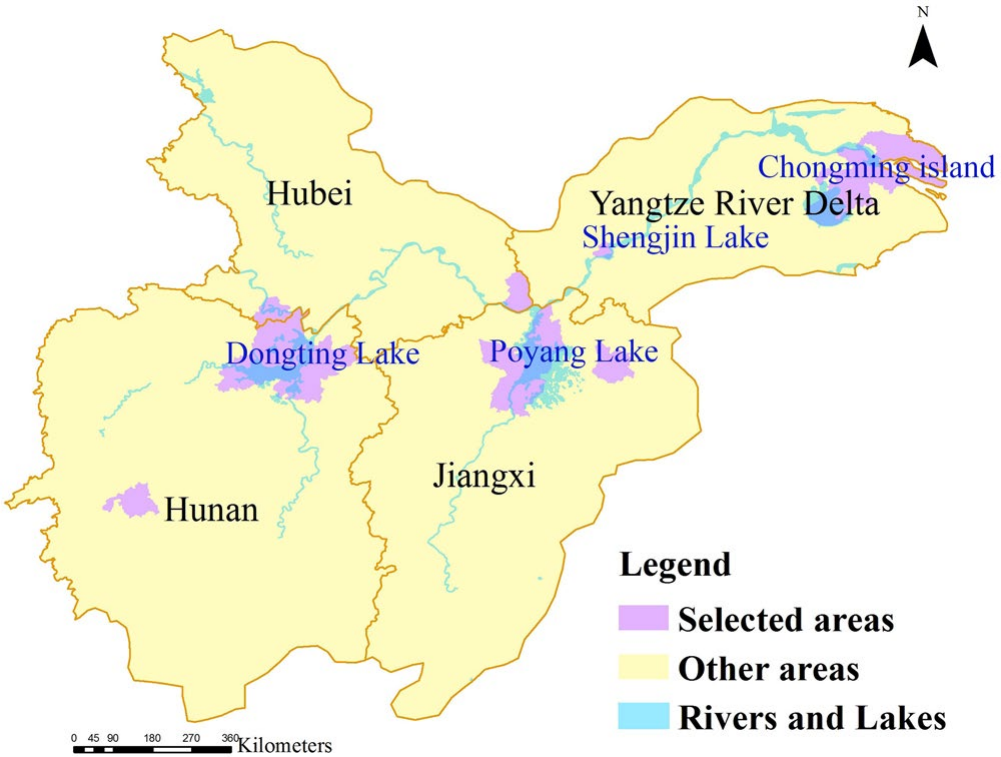
Result of the Marxan best solution for each scenario. The map was plotted using ArcGIS 10.2 (ESRI, Redlands, CA, USA, http://www.esri.com/).

Marxan selected some of the optimal conservation sites in four regions. Most of the selected sites were located in Hunan province, Hubei province and Yangtze River Delta. Figure 3 showed the distribution of these critical sites for LWfG. The most representative areas in these regions were: Dongting Lake wetland, Chongming island wetland, Shengjin Lake wetland and Poyang Lake wetland. The results represented a series of areas in which conservation measures must be taken. In spite of HSI in “Dongting Lake” and “Shengjin Lake” has decreased according to our habitat suitability models, priority reserve was still mainly distributed in here.

### MPT analyses

**Figure 4:**
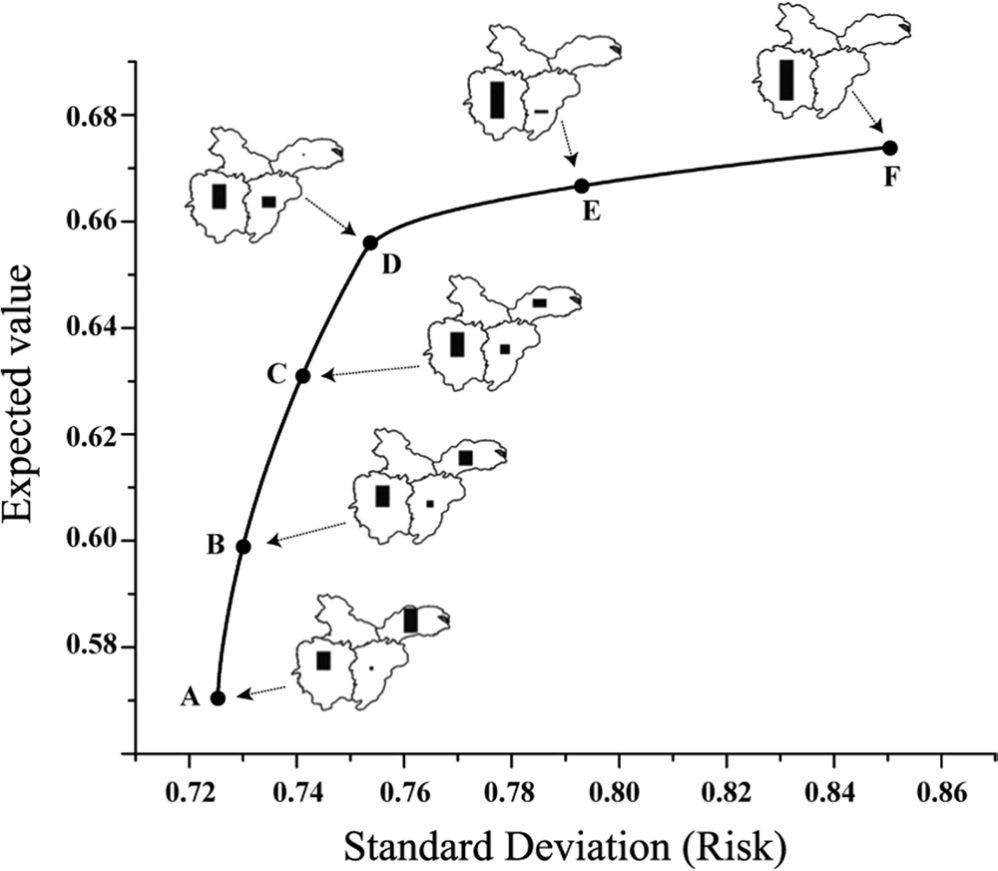
Efficient frontier of MPT analysis. Point F maximize the expect value of HSI, point A minimize the risk of uncertainty, other points are some representative points. Content for the maps was generated in ArcGIS 10.2 (ESRI, Redlands, CA, USA, http://www.esri.com/).

Efficient frontier results were obtained from MPT analysis and shown in Fig. 4. It revealed the purpose of maximizing the return and minimizing the risk, which meant to get the balance between return and risk. By finding the composition of investment portfolio weights, six portfolio points were chosen for discussion and detailed informations of these points were shown in Table 2. Portfolio with the highest expected values of HSI also had the most uncertain factor associated with their outcomes. Therefore, the efficient frontier was upward sloping shape in risk/expected benefit space. It was possible to reduce uncertainty in future conservation by accepting lower expected returns. Starting from point A to point F, expected value increased from 0.572 to 0.673 when risk increased from 0.726 to 0.843. Comparing the different portfolio weights in Table 2, we saw that the portfolio recommendations were also different. The expected return of HSI was maximized by investing in Hunan and Jiangxi regions if high conservation costs could be sustained. And, the uncertain risk could be lower when changing investment into the Yangtze River Delta region and Jiangxi sub-regions.

**Table 2:** Selected results of optimal portfolio analyses.

	Hunan	Yangtze River delta	Hubei	Jiangxi
A	0.4770	0.4961	0	0.0269
B	0.5428	0.3352	0	0.1219
C	0.6198	0.2328	0	0.1474
D	0.6809	0.0638	0	0.2553
E	0.8422	0.0444	0	0.1134
F	1	0	0	0

In these cases, we chose a series of efficient portfolios with more return and less sacrificing in the expected value. Decision makers could invest more funds in the Yangtze River Delta region to minimize the risk of uncertainty, or put more investment in Hunan and Jiangxi region to maximize the return. In this study, Hubei region was not a good choice for LWfG investment, because the portfolio weights were zero. In the history researches for LWfG, Hubei region used to be one of winter habitat for LWfG in 1990s^26–28^, but on account of the environment change, there are few LWfG in there now.

## Discussion

Maxent prediction of LWfG’s habitats showed a good consistency with historical occurrence records^24,25,29^. Besides a large number of known presence locations in Dongting Lake and Shengjin Lake, our HSM suggested that some areas in Poyang Lake, Chongming Island, Jiangsu province and Anhui province were highly suitable for LWfG. It should be noticed that HSI in the Yangtze River delta region decreased more slowly than other areas in the future (Supplementary Fig. S3), and bird survey also showed that LWfG had appeared frequently in these areas. This means that even though the cost of energy to arrive there might be higher, LWfG was still able to utilize these new habitats in the future as soon as energy intake was greater than energy expenditure^27^. *Eleocharis migoana* and *Alopecurus aequalis* were two species that meet the LWfG’s energy requirement^30^, planting these species or other alternatives can attract LWfG to spend winter here, and it might be an effective way to cope with habitat quality decreasing in Dongting Lake and Shengjin Lake.

By using MPT, conservation-outcome uncertainty can be decreased if we determined an asset allocation for the investment in Middle and Lower Reaches of the Yangtze River. By moving portfolio point from point A to point D, it could be found that risk increased along with investment shifting to Hunan and Jiangxi. From point D to point F, investment shifted from the Yangtze river delta and Jiangxi to Hunan. In other words, Hunan region is a choice for higher return, and the Yangtze River Delta region is a better selection for lower risk. Point D is “tipping points” in efficient frontier, since point C to point D has less increasement of risk than point D to point E. There is a trade-off between risk and return. Decision makers can choose portfolio weights from efficient frontier that satisfies their appetite for risk. Any portfolio weights that were not located in the frontier could be considered inefficient and the return could be maintained with more stability variance. The portfolio weights could be used by decision makers and even international animal protection organizations (eg. World Wildlife Fund (WWF, https://www.worldwildlife.org/) and BirdLife (http://www.birdlife.org/))^9,14,31,32^, and any limited resource portfolio like manpower and fund could be optimized using MPT to decrease the risk of investment.

Marxan software was especially suitable to identify the optimal regions for protection of LWfG. PA optimization increased the efficiency of management investment by pointing out the priority reserve, while MPT analyses enhanced the investment plan in quantitative terms^33^. Combining these two approaches (MPT-Marxan) was reliable since it provided the solution to risk management and priority areas selection for biodiversity conservation. It could be a useful planning tool in a wide range that had the following two advantages. First, despite climate change posed considerable uncertainty to the future habitat suitability, the influence of uncertainty could be reduced technically by using MPT to diversify the investment. Second, in order to evaluate the return and balance of alternative zoning configurations, a systematic planning framework was provided in Marxan, which was critical for the decision makers.

The combined MPT and Marxan approach provided portfolio weights for investments and guidelines to implement conservation actions. The method can be used simultaneously based on following characteristics^13^. Firstly, there should be a spatial distribution region over which the result of interest is somewhat fungible. Secondly, there should be significant uncertainty to predict future climate change, and the cost and return of conservation activity could be targeted. Thirdly, there must be or used to be conservation meaning for conservation feature(s).

At present, China’s nature reserve is in the period of the transition from quantity to quality construction^34^. Making it possible to carry out one-to-one rules to save endangered species like LWfG. Also, Systematic conservation planning (SCP) is a better method to design nature reserve^35^. It aims at designing a reserve network for all relevant species. MPT-Marxan can be utilized to guide the design of SCP based on the assumption of understanding all characters of species. It’s worth noting that the interation between species and environmental factors should be accurate definited in SCP. More complicated process should be added in conservation plans. We recommend more researches to study the relationship between species, and accurately define the uncertainty of climate change to find more proper ways to protect species.

## Method

### Study area and target species

The Yangtze River is the third longest river in the world and the longest and largest river in China. This study is conducted in the middle and lower reaches of the Yangtze River region (approximately 28°30′N-30°20′N, 111°40′E-113°10′E)^36^. The special geographical location and complex natural environment in the area leads to the abundance of bird species and ecosystem types. LWfG is mainly distributed here^37^. It has experienced a drastic decline in quantity during 20th century, and the species is now classified as Vulnerable in the IUCN Red List^38^.

### Habitat suitability models

The Maxent tool^7^ was employed and modified to predict habitat suitability for LWfG^39^. Several studies indicated that food availability influenced LWfG population distribution and abundance by affecting the physical condition, migration dates and subsequent breeding success of migrants^30,40,41^. In preliminary experiments, the AUC values of models which used plant species data and LWfG occurrence data were generally higher than that using LWfG occurrence data only. Therefore, coupling plants distribution data could improve the results of LWfG models. In our study, plants distribution data and bioclimatic variables (including current and future data) were used to build HSM to simulate plant distribution in the current and future scenarios. Then, the simulated plants distribution, land-use type, human influence variables (including the distance to roads and the distance to residences), landscape matrix (patch density) and LWfG occurrence data were selected to build HSM for LWfG^42^.

### Data access

As a fundamental factor influencing the results, species occurrence data play an important role in HSM. To assure LWfG existed in the data area, seventy-two occurrence records for LWfG were collected from wintering season synchronous survey data in 2004, 2005, 2011 and 2015, which are taken in middle and lower sections of the Yangtze River. The plants occurrence records were selected from the National Specimen Information Infrastructure (http://www.nsii.org.cn/), the Chinese Virtual Herbarium (http://www.cvh.org.cn/), the Global Biodiversity Information Facility (http://www.gbif.org/) and the literature. There are six plants relevant to LWfG’s diet selection, including *Carex heterolepis*, *Alopecurus aequalis*, *Eleocharis migoana*, *Cynodon dactylon*, *Polygonum criopolilanum*, *Carex unisexualis* ^24,25^.

Bioclimatic variables are meaningful for defining the environmental niche of species^43^. Data of nineteen bioclimatic variables (Supplementary Table S3) in current and GCM models under three representative concentration pathways scenarios (RCP 2.6 RCP 4.5 and RCP 8.5) in 2070 were downloaded from the web site of http://www.worldclim.org/. A geographical base map of middle and lower reaches of the Yangtze River region was obtained from National Fundamental Geographic Information System (http://nfgis.nsdi.gov.cn/). Land use information in 2000 and 2010 was extracted from the MODIS annual land use cover product (MOD12Q1) (Supplementary Table S4). MOD12Q1 was based on the International Geosphere-Biosphere Program (IGBP) classification system that includes a classification algorithm of decision tree and artificial neural network. It was an important data source and has extensively applied to monitor Land-Use and Land-Cover Change (LUCC) dynamics. The data were divided into 16 classes by land use, and the resolution was transformed to 30 seconds. FRAGSTATS (version 4.2) was used to calculate the patch area. This data could be exported to ArcGIS 10.2 to calculate the patch density through “Zonal statistics” tool. Distance to residents and distance to roads were calculated by Inverse Distance Weighted (IDW) interpolation. All of these variables were transformed into “asc” format by ArcGIS 10.2.

### PA optimization

Marxan optimized a set of locations to meet the requirement of conservation target with minimum cost. This method used the following parameters^44^:

1. Planning units (PUs)
  Because of computational capabilities, we up-scaled the resolution of grid cells to 2.5′ × 2.5′. This step resulted in 9360 planning units covering the whole Middle and Lower Reaches of the Yangtze River. Each one had an unique identification code.
  The selection of nature reserves was based on existing data from State Environment Protection Administration (http://www.mep.gov.cn/stbh/), and only national and provincial birds nature reserves were considered in this research (Supplementary Table S5).
2. Targets of consrevation
  According to LWfG’s situation, the target of conservation was set by the IUCN Red List categories, the targets of vulnerable species was assigned for 20%.
3. Costs

Three factors could be included as relative cost indicators^45^: (1) ecological disturbance, which represented the level of human disturbance within a particular habitat, (2) opportunity cost, which standed for the profit would be gained when the area were not converted into nature reserves, (3) start-up cost, money consumption to converte these area into nature reserves. Considering the low value of “ecological disturbance” and “opportunity cost” in these areas with high “start-up cost”, the cost of each planning unit is seted as 10,000/(HSI)^22^, i.e. it is considered to be more efficient to conserve the areas with high richness of LWfG. HSI calculated from RCP 4.5 scenario was used for this calculation.

Marxan aimed to find an objective function (optimal solution for nature reserves) that included the cost of the selected sites and additional penalty (Eq. 3). The boundary length modifier (BLM) added weighted importance relative to the other components of the objective. Species Penalty Factor (SPF) weighted species penalty if target for the LWfG was not met.

1 $${\rm{Objective}}\,{\rm{function}}=\sum _{{\rm{restoration}}\,{\rm{sites}}}{\rm{Cost}}+{\rm{SPF}}\times {\rm{Penalty}}+{\rm{BLM}}\times {\sum }^{}\,{\rm{Boundary}}$$

Model had been run 100 times totally to calculated the “selection frequency” for each planning units. Grid cells with more than 80 times were selected as the priority areas for conservation^46^.

### Portfolio analyses

In MPT analyses, mean-variance was used to depict the return and risk. “Mean” represented expected return in portfolio, and it was defined as the weighted average of expected return. “Variance” meant the standard deviation of the expected returns (the portfolio’s volatility), and it described risk in investment^47^. According to historic researches of MPT in ecology to portfolio weights^6,9,13,14,48^, taking the average value from HSM or SDM was the only way to get expected return, and it has been shown to be correct^6,9^.

However, decision makers are not allowed to allocate the investment to the whole area due to funding limitations. Allocating the investment to existing nature reserve for birds is a better choice for them. Therefore, we applied Marxan to help decision makers choosing the key area in this research. Average value of HSI in area that Marxan selected was used for MPT analyses to get expected return.

We defined the return as *R* = *HSI*, so the expected return for each analysis was defined as

2 $${\rm{E}}({HS}{{I}}_{i})=\sum _{j}\,{P}_{j}\times {HS}{{I}}_{{ij}}\,{\rm{for}}\,{\rm{all}}\,{\rm{climate}}\,{\rm{scenarios}}\,{\rm{j}}$$

*E(HSI)* refered to the expected HSI, equalled to the sum of probabilities of each climate scenario (current, RCP2.6, RCP4.5, RCP8.5. In this research set probabilities as 0.25 for each scenarios, meant each scenario had the same probability^48,49^) timed the average HSI in area that Marxan selected in year *i* for climate scenario *j* (see Supplementary Table S6).

The aim of MPT analysis was to minimize the risk of portfolios for a specific expected HSI by selecting the elements of the portfolio appropriately^50,51^. Formally,

3 $${\rm{\min }}\,{w}^{T}\sum {\rm{w}},\,{\rm{subject}}\,{\rm{to}}\,\sum _{i}\,{w}_{i}=1,\,{w}_{i}\ge {\rm{0}}\,{\rm{for}}\,{\rm{all}}\,i,\,{\rm{and}}\,{E}{(R)}^{T}\,w=\mu \,(R={HSI})$$

where *w*_*i*_ were the weights of the portfolio of investment in our study area, *T* was the transpose operator, ${w}^{T}\sum w$ was the covariance matrix of portfolio asset returns, *i* represented potential assets in the portfolio, and *R* was defined as HSI in this paper. *μ* referred to the expected return.

Tracing the mean return and variance intersections, it had been drawn a continuous curve called efficient frontier in the Markowitz theory. Every point in efficient frontier represented one optimum portfolio, which meant the best possible expected level of return in its level of risk^52^. Frontcon routine in the Financial Toolbox of the Matlab R2011a release was used to analyse the data^48^.

### Data availability

The datasets analysed during the current study are available from the corresponding author on reasonable request.

## Electronic supplementary material

Supplementary Information

Dataset 1
