# Supplementary material for: Coupling Modern Portfolio Theory and Marxan enhances the efficiency of Lesser White-fronted Goose’s (Anser erythropus) habitat conservation
Source: Sci Rep. 2018 Jan 9;8:214. doi: 10.1038/s41598-017-18594-2 (PMC5760730; doi:10.1038/s41598-017-18594-2)
Supplement: Supplementary file 1 — Supplementary Information [file 41598_2017_18594_MOESM1_ESM.doc]

**Coupling Modern Portfolio Theory and Marxan enhances the efficiency of Lesser White-fronted Goose’s (*Anser erythropus*) habitat conservation**

Jie Liang^1,2^*, Xiang Gao^1,2^, Guangming Zeng^1,2^*, Shanshan Hua^1,2^, Minzhou Zhong^1,2^, Xiaodong Li^1,2^, Xin Li ^1,2^

^1^ College of Environmental Science and Engineering, Hunan University, Changsha 410082, PR China

^2^ Key Laboratory of Environmental Biology and Pollution Control (Hunan University), Ministry of Education, Changsha 410082, PR China

***Corresponding author:**

Jie Liang*, College of Environmental Science and Engineering, Hunan University, Changsha 410082. E-mail: liangjie82@163.com and liangjie82@163.com; Guangming Zeng*, College of Environmental Science and Engineering, Hunan University, Changsha 410082. E-mail: zgming@hnu.edu.cn

**Table S1 AUC value of plants HSM and LWfG HSM**

| **species** | **AUC value** |
| --- | --- |
| *Carex heterolepis* | 0.911 |
| *Heleocharis migoana* | 0.901 |
| *Alopecurus aequalis* | 0.900 |
| *Cynodon dactylon* | 0.872 |
| *Polygonum criopolilanum* | 0.867 |
| *Eleocharis valleculosa* | 0.880 |
| LWfG^a^ | 0.887 |
| LWfG^b^ | 0.891 |
| LWfG^c^ | 0.806 |
| LWfG^d^ | 0.812 |
| LWfG^e^ | 0.981 |
| LWfG^f^ | 0.976 |
| LWfG^g^ | 0.988 |
| LWfG^h^ | 0.965 |

^a^ Habitat suitability model in current situation with bioclimatic variables

^b^ Habitat suitability model in RCP 2.6 situation with bioclimatic variables

^c^ Habitat suitability model in RCP 4.5 situation with bioclimatic variables

^d^ Habitat suitability model in RCP 8.5 situation with bioclimatic variables

^e^ Habitat suitability model in current situation with plants distribution variables

^f^ Habitat suitability model in RCP 2.6 situation with plants distribution variables

^g^ Habitat suitability model in RCP 4.5 situation with plants distribution variables

^h^ Habitat suitability model in RCP 8.5 situation with plants distribution variables

**Table S2** Relative contributions of the environmental variables to the Maxent model for LWfG in current scenario. To determine the first estimate, in each iteration of the training algorithm, the increase in regularized gain is added to the contribution of the corresponding variable, or subtracted from it if the change to the absolute value of lambda is negative. For the second estimate, for each environmental variable in turn, the values of that variable on training presence and background data are randomly permuted. The model is reevaluated on the permuted data, and the resulting drop in training AUC is shown in the table, normalized to percentages.

| **Variable** | **Percent contribution** | **Permutation importance** |
| --- | --- | --- |
| *Eleocharis* *migoana* | 44 | 52.7 |
| *Alopecurus* *aequalis* | 20 | 14.8 |
| *Carex* *heterolepis* | 15.9 | 15.3 |
| Land use | 10.6 | 9.6 |
| Patch density | 6.1 | 1.3 |
| *Cynodon* *dactylon* | 2.3 | 4.1 |
| *Polygonum* *criopolilanum* | 0.5 | 1.2 |
| *Carex unisexualis* | 0.4 | 1.1 |
| distance to road | 0.1 | 0 |
| distance to residents | 0 | 0 |

**Table S3** Environmental variables used for building LWfG’s HSM

| Code | Environmental variables | Unit |
| --- | --- | --- |
| Bio1 | Annual mean temperature | °C |
| Bio2 | Mean diurnal range (mean of monthly (max–min temp)) | °C |
| Bio3 | Isothermality ((Bio2/Bio7) × 100) |  |
| Bio4 | Temperature seasonality (standard deviation × 100) | C of V |
| Bio5 | Maximum temperature of warmestmonth | °C |
| Bio6 | Minimum temperature of coldest month | °C |
| Bio7 | Temperature annual range (Bio5–Bio6) | °C |
| Bio8 | Mean temperature of wettest quarter | °C |
| Bio9 | Mean temperature of driest quarter | °C |
| Bio10 | Mean temperature of warmest quarter | °C |
| Bio11 | Mean temperature of coldest quarter | °C |
| Bio12 | Annual precipitation | mm |
| Bio13 | Precipitation of wettest period | mm |
| Bio14 | Precipitation of driest period | mm |
| Bio15 | Precipitation seasonality (coefficient of variation) | C of V |
| Bio16 | Precipitation of wettest quarter | mm |
| Bio17 | Precipitation of driest quarter | mm |
| Bio18 | Precipitation of warmest quarter | mm |
| Bio19 | Precipitation of coldest quarter | mm |
|  | Patch density | normalization processing |
|  | Distance to residents | m |
|  | Distance to road | m |
|  | Land use |  |

**Table S4** Land Cover Types Description from the MODIS Land Cover Type product (MCD12Q1)

| Class | Type |
| --- | --- |
| 0 | Water |
| 1 | Evergreen Needleleaf forest |
| 2 | Evergreen Broadleaf forest |
| 3 | Deciduous Needleleaf forest |
| 4 | Deciduous Broadleaf forest |
| 5 | Mixed forest |
| 6 | Closed shrublands |
| 7 | Open shrublands |
| 8 | Woody savannas |
| 9 | Savannas |
| 10 | Grasslands |
| 11 | Permanent wetlands |
| 12 | Croplands |
| 13 | Urban and built-up |
| 14 | Cropland/Natural vegetation mosaic |
| 15 | Snow and ice |
| 16 | Barren or sparsely vegetated |

**Table S5** Current wetland natural reserve in the Middle and Lower reaches of Yangtze River (download from website of Ministry of Environmental Protection of the People's Republic of China: <http://sts.mep.gov.cn/zrbhq/zrbhq/>)

| Name | Location | Main protection objects |
| --- | --- | --- |
| Eastern Hongze Lake Wetland Natural Reserve | Hongze County, Huai'an City | Lacustrine wetland ecosystem and rare birds |
| Gaoyou Lake Wetland Natural Reserve | Gaoyou City | Wetland ecosystem |
| Luoma Lake Wetland Natural Reserve | Suqian City | Wetland ecosystem and birds |
| Sifang Lake Wetland Natural Reserve | Huaiyuan County | Wetland ecosystem and rare water birds |
| Tuohu Lake Natural Reserve | Wuhe County | Wetland ecosystem and bird |
| Shijiu Lake Natural Reserve | Dangtu County | Wetland ecosystem and rare water birds |
| Nvshan Lake Natural Reserve | Mingguang City | Wetland ecosystem and the water and aquatic living beings |
| Shibasuo Wetland | Chizhou City | Wetland ecosystem and rare water bird |
| Yaohu Lake Natural Reserve | Nanchang County | Winter migrant birds and wetland ecosystem |
| Poyang Nanji Wetlands Natural Reserve | Xinjian County | Winter migrant birds and wetland ecosystem |
| Hexi wetland of Poyang Lake | Yongxiu County | Wetland ecosystem and migrant birds |
| Pingfeng wetland | Hukou County | Wetland ecosystem and migrant birds |
| Gongqing south lake wetland | Gongqingcheng City | Wetland ecosystem and migrant birds |
| Chenhu wetland | Wuhan City | Wetland ecosystem and migrant birds |
| Wanghu Wetland Nature Reserve | Yangxin County | Fresh water lake wetland ecosystem and birds |
| Eastern Dongting Lake Nature Reserve | Yueyang City | Wetland ecosystem and rare water birds |
| South Dongting Lake Nature Reserve | Yiyang City | Wetland ecosystem and rare water birds |
| Hengling lake nature Reserve | Xiangyin county | Wetland ecosystem and rare birds |
| Chongming Dongtan Birds Nature Reserve | Shanghai City | Wetland ecosystem and rare birds |

**Table S6**. Average value of HSI value in area that Marxan selected for LWfG

| Region | Current | RCP 2.6 | RCP 4.5 | RCP8.5 |
| --- | --- | --- | --- | --- |
| Hunan | 0.873 | 0.655 | 0.716 | 0.613 |
| Hubei | 0.714 | 0.533 | 0.592 | 0.631 |
| Yangtze River delta | 0.778 | 0.643 | 0.692 | 0.656 |
| Jiangxi, | 0.747 | 0.686 | 0.626 | 0.641 |


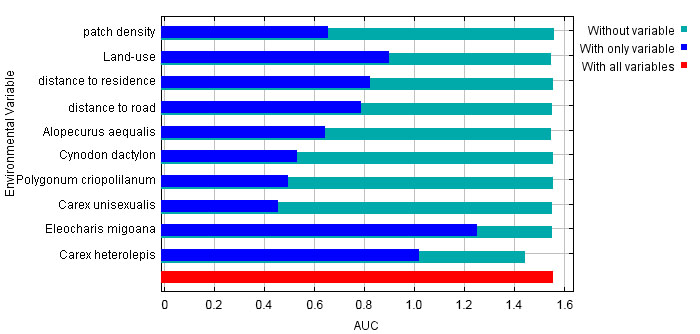


**Fig. S1** The results of the jackknife test of variables’ contribution in modeling LWfG’s HSM in current situation. (The regularized training gain describes the level that the Maxent distribution fits the presence data compared to a uniform distribution. The dark blue bars indicate that the gain from using each variable in isolation, the light blue bars indicate the gain lost by removing the single variable from the full model, and the red bar indicates the gain using all of the variables.)


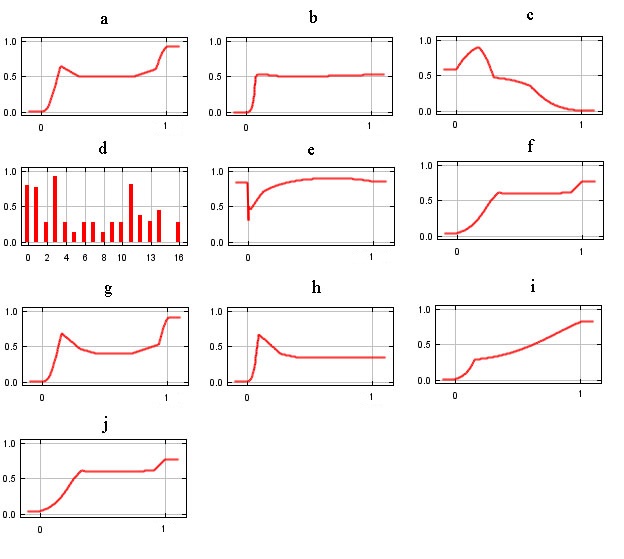
**Fig. S2** Response curves of 10 environmental variables in modeling LWfG’s HSM in current situation. (a: HSI of *Carex* *heterolepis*, b: HSI of *Alopecurus* *aequalis*, c: HSI of *Eleocharis* *migoana*, d: Land use, e: distance to road, f: HSI of *Cynodon* *dactylon* g: HSI of *Polygonum* *criopolilanum*, h: HSI of *Carex unisexualis*, i: Patch density, j: distance to residents)


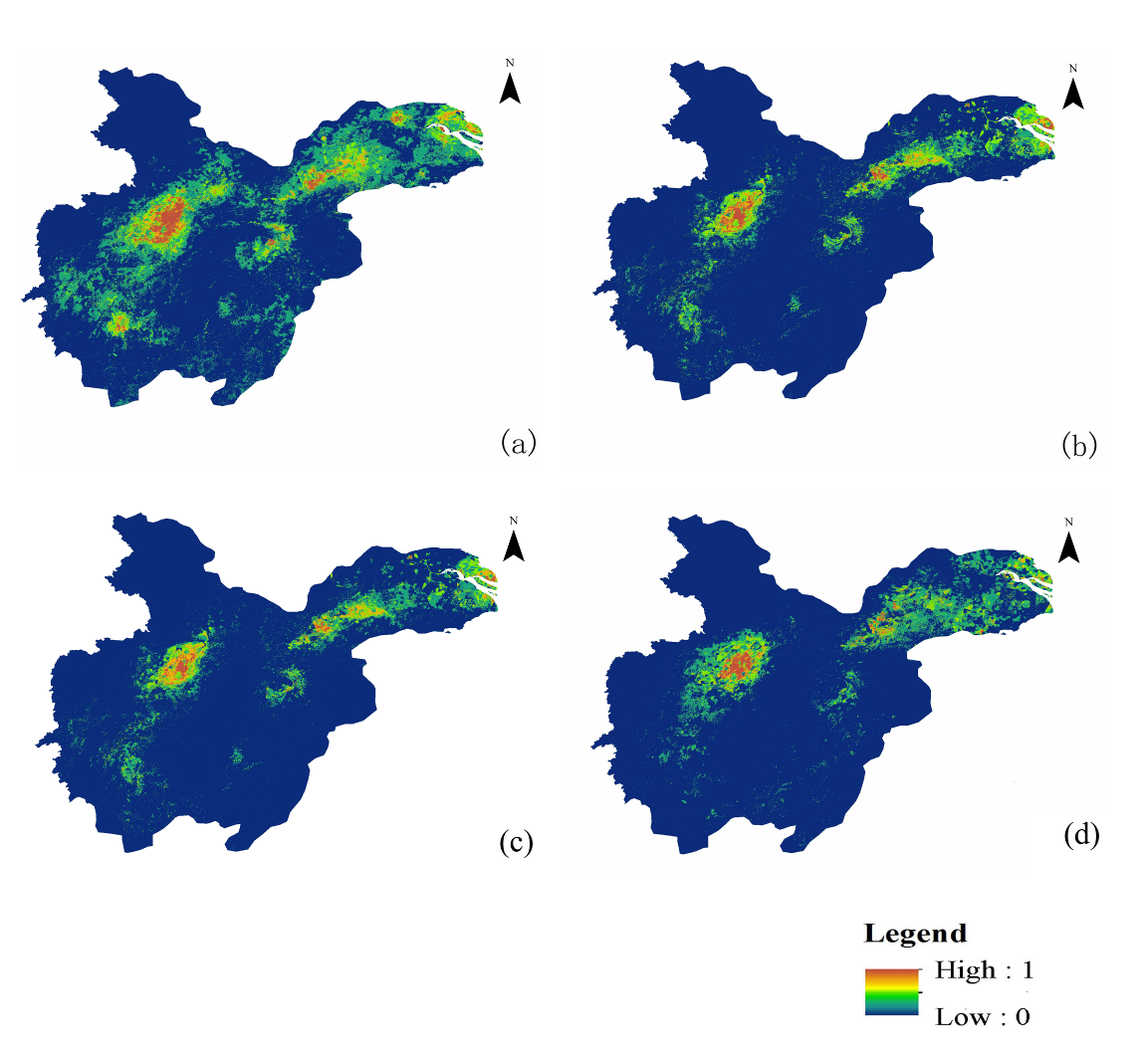


**Fig. S3** Map of habitat suitability index in different scenarios. (a) current (b) RCP 2.6 (c) RCP4.5 (d)RCP8.5. All maps were plotted using ArcGIS 10.2 (ESRI, Redlands, CA, USA, http://www.esri.com/).
